# Elimination of protein aggregates prevents premature senescence in human trisomy 21 fibroblasts

**DOI:** 10.1371/journal.pone.0219592

**Published:** 2019-07-29

**Authors:** Nobutoshi Nawa, Katsuya Hirata, Keiji Kawatani, Toshihiko Nambara, Sayaka Omori, Kimihiko Banno, Chikara Kokubu, Junji Takeda, Ken Nishimura, Manami Ohtaka, Mahito Nakanishi, Daisuke Okuzaki, Hidetoshi Taniguchi, Hitomi Arahori, Kazuko Wada, Yasuji Kitabatake, Keiichi Ozono

**Affiliations:** 1 Department of Pediatrics, Graduate School of Medicine, Osaka University, Suita, Osaka, Japan; 2 Department of Neonatal Medicine, Osaka Women’s and Children’s Hospital, Izumi, Osaka, Japan; 3 Department of Pediatrics, Indiana University School of Medicine, Indianapolis, Indiana, United States of America; 4 Department of Genome Biology, Graduate School of Medicine, Osaka University, Suita, Osaka, Japan; 5 Laboratory of Gene Regulation, Faculty of Medicine, University of Tsukuba, Tsukuba, Ibaraki, Japan; 6 Biotechnology Research Institute for Drug Discovery, National Institute of Advanced Industrial Science and Technology (AIST), Tsukuba, Ibaraki, Japan; 7 Genome Information Research Center, Research Institute for Microbial Diseases, Osaka University, Suita, Osaka, Japan; Institut du cerveau et de la moelle epiniere, FRANCE

## Abstract

Chromosome abnormalities induces profound alterations in gene expression, leading to various disease phenotypes. Recent studies on yeast and mammalian cells have demonstrated that aneuploidy exerts detrimental effects on organismal growth and development, regardless of the karyotype, suggesting that aneuploidy-associated stress plays an important role in disease pathogenesis. However, whether and how this effect alters cellular homeostasis and long-term features of human disease are not fully understood. Here, we aimed to investigate cellular stress responses in human trisomy syndromes, using fibroblasts and induced pluripotent stem cells (iPSCs). Dermal fibroblasts derived from patients with trisomy 21, 18 and 13 showed a severe impairment of cell proliferation and enhanced premature senescence. These phenomena were accompanied by perturbation of protein homeostasis, leading to the accumulation of protein aggregates. We found that treatment with sodium 4-phenylbutyrate (4-PBA), a chemical chaperone, decreased the protein aggregates in trisomy fibroblasts. Notably, 4-PBA treatment successfully prevented the progression of premature senescence in secondary fibroblasts derived from trisomy 21 iPSCs. Our study reveals aneuploidy-associated stress as a potential therapeutic target for human trisomies, including Down syndrome.

## Introduction

Down syndrome (DS; trisomy 21) is the most common chromosomal abnormality, affecting 1 in 650–1000 births [1]. Most cases of DS have an extra copy of chromosome 21, exhibiting various types of clinical complications including intellectual disability, congenital heart defects and hematopoietic abnormalities. These phenotypes are generally thought to be a direct result of cumulative effects caused by increased expression of a specific subset of genes located on chromosome 21. Intensive studies have been made to identify the combination of genes responsible for disease phenotypes, providing clues to decipher the molecular consequences of genome dosage imbalances. Many features, such as pediatric leukemia in DS, can be clearly explained by this “gene dosage effects” hypothesis, and several candidate genes have been identified using cell and animal models [2–4]. However, the clinical presentation of DS is complex and highly variable, and there seems not always to be a direct correlation between gene dosage and disease phenotypes, suggesting the existence of different mechanisms that can modify the gene-specific effect and have a strong impact on DS pathology.

It is commonly accepted that organismal aneuploidy causes growth defects in plants [5], or embryonic lethality and developmental impairment in metazoans, across species [6, 7]. Studies on whole-chromosome gains in budding yeast clearly showed that aneuploidy exerted a proliferation defect regardless of the origin of the extra chromosome, and the severity of the phenotype tended to scale with the degree of deviation from the euploid karyotype [8–10]. Intriguingly, this impaired proliferation effect was attributed to the karyotypic alteration itself, that is, to the cumulative effects of many genes that confer no observable phenotype individually, rather than to the specific effects of a few dosage-sensitive genes on the extra chromosome [11]. Meta-analysis of gene/protein expression data from aneuploid cells in diverse organisms has revealed a novel aneuploidy-associated expression signature characteristic of altered metabolism and stress response [12, 13]. Subsequent studies on budding yeast have demonstrated that chromosome aneuploidy causes a variety of cellular stresses, including genomic instability, cell-cycle arrest, and proteotoxic and oxidative stresses, which are described as aneuploidy-associated stresses [14, 15].

In humans, aneuploidy occurs in more than 5% of pregnancies. Most of these terminate *in utero*, and trisomy 21, 18 and 13 are the only autosomal trisomies that are compatible with livebirth [16]. Among these syndromes, most patients with trisomies 18 and 13 cannot survive beyond the first year of life, but average life expectancy for trisomy 21 is around 60 years [17, 18]. Importantly, chromosomes 21, 18 and 13 are those which contain the least number of protein-coding genes, and there seems to be a correlation between life expectancy or the severity of clinical symptoms and the number of coding genes on a trisomic chromosome. These results strongly suggest that recognizable phenotypes in individuals with trisomy can be formed by the synergistic interaction between chromosome-specific effects of dosage-sensitive genes and aneuploidy-associated stress.

Given the potential roles of aneuploidy-associated stress in human disease, it is critical to understand whether and how abnormal karyotypes affect cellular physiology in human primary cells. Here, we analyzed the physiology of human primary fibroblasts derived from patients with trisomies 21, 18 and 13. All of the trisomy cell lines showed impaired proliferation, as well as markedly increased populations of senescence-associated beta-galactosidase (SA-β-gal)-positive cells. Senescent trisomy fibroblasts contained excess amounts of total RNA and protein, accompanied by a widespread increase of transcription. This amplified transcription and protein synthesis caused an alteration of energy metabolism, leading to an excessive production of reactive oxygen species (ROS), mitochondrial damage, and the accumulation of protein aggregates, indicating that human trisomies exert common physiological stress. We found that sodium 4-phenylbutyrate (4-PBA), a potent chemical chaperone compound, was able to reduce the accumulation of protein aggregates in trisomy fibroblasts. In addition, the progression of premature senescence induced by trisomy could be successfully suppressed by 4-PBA treatment in trisomy 21 induced pluripotent stem cell (iPSC)-derived secondary fibroblasts.

## Materials and methods

All *in vitro* and *in vivo* studies were approved by the Ethics Committee and Animal Experimental Faculty of Osaka University Graduate School of Medicine.

### Cell counting

On day 0, 3×10^4^ cells were seeded into culture plates. Three days after seeding, the number of cells was counted after detaching them using trypsin-EDTA solution (0.25% (w/v) trypsin, 1 mmol/l ethylenediaminetetraacetic acid (EDTA)-4Na solution; FUJIFILM Wako Pure Chemical Corporation, Osaka, Japan).

### Apoptosis assay

A terminal deoxynucleotidyl transferase-mediated dUTP nick end labeling (TUNEL) assay was used to assess apoptosis, with an In situ Apoptosis Detection Kit (Takara Bio Inc., Siga, Japan), in accordance with the manufacturer’s instructions. Nuclei were counterstained with Hoechst (Dojindo, Kumamoto, Japan). TUNEL-positive cells and total cell number in a field of view were automatically counted by an IN Cell Analyzer 6000 (GE Healthcare, Little Chalfont, UK), and the positive ratio was calculated.

### SA-β-gal assay

A Senescence β-Galactosidase Staining Kit (Cell Signaling Technology, Inc., Danvers, MA, USA) was used to assess cellular senescence, in accordance with the manufacturer’s instructions. Nuclei were counterstained with Hoechst. SA-β-gal-positive cells and total cell numbers in a field of view were automatically counted by an IN Cell Analyzer 6000, and the positive ratio was calculated. For assessing the effects of different chemical compounds on cellular senescence, 3×10^4^ cells were seeded on day 0, cultured with each compound for two weeks, and the SA-β-gal-positive cell ratio was then calculated. The compounds used in these analyses included 2 mM sodium 4-PBA, (Sigma-Aldrich, St. Louis, MO, USA), and 2.5 mM N-Acetyl-L-cysteine (NAC, Sigma-Aldrich).

### ROS assay

The level of ROS was assessed as described previously [19]. Briefly, mitochondrial superoxide was quantified by staining with 5 μM MitoSox red (Thermo Fisher Scientific Inc., Waltham, MA, USA) for 15 minutes. Mitochondria were stained with 100 nM Mitotracker Green FM (Thermo Fisher Scientific Inc.) for 15 minutes. The MitoSox/Mitotracker signal ratio was used to assess the ROS level per mitochondria. All images were collected with an IN Cell Analyzer 6000.

### Mitochondrial membrane potential

Mitochondrial membrane potential was assessed with a MITO-ID Membrane potential detection kit (Enzo Life Sciences Inc., Farmingdale, NY, USA), in accordance with the manufacturer’s instructions. All images were collected with an IN Cell Analyzer 6000.

### Aggresome assay

A PROTEOSTAT Aggresome Detection kit (Enzo Life Sciences Inc.) was used to detect aggresomes in cells, in accordance with the manufacturer’s instructions. Nuclei were counterstained with Hoechst. Cytoplasm was stained with HCS CellMask Deep Red Stain (Thermo Fisher Scientific Inc.). All images were collected with an IN Cell Analyzer 6000. To assess the effects of different chemical compounds on aggregate accumulation, cells were cultured with each compound and aggregate intensities were evaluated. The compounds used in these analyses included trimethylamine N-oxide (TMAO, Sigma-Aldrich), sodium 4-PBA, Sigma-Aldrich), geldanamycin (TCI, Tokyo, Japan), 2-hydroxypropyl-β-cyclodextrin (Sigma-Aldrich), rapamycin (Sigma-Aldrich), and valproic Acid (VPA, FUJIFILM Wako Pure Chemical Corporation).

### Western blotting

Western blotting was performed as described previously, with some modifications [20]. Briefly, cells were lysed with RIPA Buffer (FUJIFILM Wako Pure Chemical Corporation) containing a protease inhibitor mixture (Roche Diagnostics, Basel, Switzerland). Equal amounts of protein (10 μg) were electrophoresed using 10% sodium dodecyl sulfate-polyacrylamide gels. Proteins were transferred to polyvinylidene difluoride membranes, washed with Tris-buffered saline containing 0.05% Triton X-100, and incubated with BlockingOne solution (Nacalai Tesque, Kyoto, Japan) for 60 minutes. Rabbit anti Phospho-Rb (1:1,000; Cell Signaling Technology, Inc.) was used as the primary antibody. Horseradish peroxidase-conjugated anti-rabbit IgG antibody (1:5,000; Promega, Madison, WI, USA) was used as the secondary antibody. As a control, β-actin was detected with the rabbit anti-β-Actin pAb-HRP-DirecT (1:2,000; MBL). Blots were visualized using Chemi-Lumi One L (Nacalai Tesque).

### Immunocytochemistry

Immunocytochemistry was performed as described previously, with some modifications [20]. Mitochondria were stained by exposing cells to MitoTracker Red CMXRos (100 nM, Thermo Fisher Scientific Inc.) for 15 minutes before fixation with 4% paraformaldehyde/phosphate-buffered saline (PBS). Cells were then permeabilized with 0.2% Tween 20/PBS for 15 minutes. Next, cells were blocked with 5% fetal bovine serum (FBS)/PBS for 30 minutes. Cells were then incubated at 4°C for 16 hours with rabbit anti-TOM20 (1:100; Santa Cruz Biotechnology, Dallas, TX, USA) primary antibody. After washing with PBS, cells were incubated for 60 minutes with goat anti-rabbit secondary antibody conjugated with Alexa Fluor 488 (1:200; Thermo Fisher Scientific Inc.). Nuclei were counterstained with Hoechst (Dojindo). Cytoplasm was stained with HCS CellMask Deep Red Stain (Thermo Fisher Scientific Inc.). All images were collected with an IN Cell Analyzer 6000.

### Isolation and culture of primary human dermal fibroblasts

Primary human dermal fibroblasts were isolated from skin biopsy specimens of four trisomy 21 patients, three trisomy 18 patients, and one trisomy 13 patient as described previously [21]. In addition, three healthy dermal fibroblast cell lines were purchased from Lonza (Walkersville, MD, USA), and Thermo Fisher Scientific Inc. (full details of the samples used in this study are provided in S1 Table). For all procedures, informed consent was obtained from each patient’s guardians in accordance with the regulations of the Ethics Committee of Osaka University Hospital. After fibroblasts grew out from the skin specimens and became subconfluent, the cells were passaged into a new dish (passage 1). Cells were passaged when the culture reached approximately 90% confluence. Primary dermal fibroblasts were maintained in Dulbecco’s modified Eagle’s medium (DMEM) containing 10% FBS (v/v), 2 mM L-glutamine, 100 U/ml penicillin and 100 μg/ml streptomycin [21]. Experiments were conducted before the passage number reached 10. Karyotype analysis confirmed that all primary trisomy fibroblasts retained their original trisomy karyotypes (S1A–S1C Fig).

### Maintenance and secondary fibroblast-like cell differentiation of human iPSCs

Human iPSCs were cultured as previously described [2, 22]. In brief, iPSCs were maintained on mitomycin C (Sigma)-inactivated mouse embryonic fibroblasts in human ES cell medium consisting of DMEM/F12 (Wako) supplemented with 20% KnockOut Serum Replacement (Gibco), 2 mM L-alanyl-L-glutamine (Wako), 1% MEM nonessential amino acid solution (Wako), 0.1 mM 2-mercaptoethanol (Sigma), and 5 ng/mL basic fibroblast growth factor (Katayama Chemical). Cultures were passaged every 6–8 days either manually or enzymatically using dispase II (Roche, Basel, Switzerland).

Secondary fibroblast-like cells were differentiated as described previously, with some modifications [23, 24]. Briefly, embryoid bodies made from human iPSCs were cultured for 4 days in nonadherent cell culture plates in differentiation medium (80% knockout DMEM (KO-DMEM; Thermo Fisher Scientific Inc.), 1 mM L-glutamine, 0.1 mM β-mercaptoethanol, 20% FBS, and 1% nonessential amino acids.) Next, the cell aggregates were seeded into gelatin-coated plates and cultured for an additional 9 days. The outgrowing cell population was used as secondary fibroblast-like cells after at least two passages, and cultured in the medium described above. One clone each of a trisomy 21 fibroblast-derived human iPSC line (Tri21 iPSCs) and corrected disomy 21 iPSC line (cDi21 iPSC) was used in the experiments.

### Protein synthesis

The Click-iT AHA Alexa Fluor 488 Protein Synthesis HCS Assay Kit (Thermo Fisher Scientific Inc.) was used to assess protein synthesis, in accordance with the manufacturer’s instructions. Briefly, cells were cultured for 30 minutes in L-methionine-free medium containing an amino acid analog of methionine with an azido moiety, and were then fixed. The amino acid analog was incorporated into proteins during protein synthesis and could be detected using the “click” reaction. All images were collected with an IN Cell Analyzer 6000, and the relative level of protein synthesis was analyzed using the IN Cell Developer Toolbox 1.9 software.

### Metabolite assays for glucose and lactate

On day 0, 1×10^5^ cells were seeded into culture plates, and the concentrations of glucose and lactate in the medium were measured using an ABL800 Flex (Radiometer, Copenhagen, Denmark). Three days after seeding, the concentrations of glucose and lactate in the medium were measured again using the same instrument, and the changes from day 0 were calculated. The results were normalized to the number of cells.

### Measurement of RNA and protein amounts in cells

On day 0, 1×10^5^ cells were seeded into 10-cm dishes. Three days after seeding, without changing the medium, the number of cells was counted after detaching cells from the culture plate using trypsin-EDTA solution (0.25% (w/v) trypsin, 1 mmol/l EDTA-4Na solution, FUJIFILM Wako Pure Chemical Corporation). RNA or protein was extracted from 1×10^5^ cells using NucleoSpin RNA II (Macherey-Nagel, Oensingen, Switzerland) or DC protein assay kits (Bio‐Rad Laboratories, Hercules, CA, USA), and the total amounts of RNA and protein in 1×10^5^ cells were determined.

### Adenosine triphosphate (ATP) concentration

Cellular ATP concentration was determined using an intracellular ATP determination kit (Toyo B-Net, Tokyo, Japan). To assess changes in intracellular ATP following inhibition of ATP production, the production was inhibited by oligomycin (2 μM, Cell Signaling Technology) for 60 minutes and the associated decreases in intracellular ATP levels were determined using the intracellular ATP determination kit (Toyo B-Net). The results were normalized to the number of cells.

### Respiration and acidification rate measurements

The respiration and acidification rates of cells were measured using an XF96 Extracellular Flux Analyzer (Seahorse Bioscience, North Billerica, MA, USA) in accordance with the manufacturer’s instructions. Briefly, 2×10^4^ cells were seeded into culture plates in culture medium a day before measurement. The assay was performed in unbuffered DMEM supplemented with 25 mM glucose, 2 mM glutamate, and 1 mM pyruvate, in accordance with the manufacturer’s instructions. Cells were preincubated in this assay medium for 1 hour before measurement. Oxygen consumption values and acidification rates were calculated in accordance with the manufacturer’s instructions.

Changes in oxygen consumption following specific inhibition of cellular processes such as RNA synthesis or protein synthesis were assessed as described previously [25]. Briefly, the oxygen consumption rate was monitored over time under baseline conditions as well as in response to the addition of inhibitors such as actinomycin D (1 μg/ml, Sigma-Aldrich) or cycloheximide (0.5 μg/ml, Sigma-Aldrich). The results were normalized to the number of cells.

### Gene expression assay using NanoString nCounter

Gene expression assays using the nCounter GX Human Cancer Reference Kit (NanoString Technologies, Seattle, WA, USA) were conducted in accordance with the manufacturer’s instructions, following the Cell Lysate Protocol. Briefly, 1×10^5^ cells were collected and lysed using 50 μl of Buffer RLT (QIAGEN, Hilden, Germany). The cell lysate (4 μl) was hybridized with the Reporter CodeSet (nCounter GX Human Cancer Reference Kit) and the Capture ProbeSet, and was processed in accordance with the instructions. Data were normalized using positive and negative controls that are included in the kit.

### Microarrays

For microarray analysis without RNA spike-in controls [26], 50 ng of total RNA was used, while for analysis with RNA spike-in controls, 100 ng of total RNA containing RNA spike-in controls (ERCC ExFold RNA Spike-In Mixes, Thermo Fisher Scientific Inc.) was used. The quality of the total RNA samples was assessed with an Agilent 2100 Bioanalyzer (Agilent Technologies, Santa Clara, CA, USA). Each total RNA sample was reverse-transcribed, and the resulting cDNA was subjected to *in vitro* transcription with T7 RNA polymerase to be labeled with Cy3. Cy3-labeled cRNAs at 1,650 ng (for analysis without RNA spike-in controls) or 600 ng (for analysis with RNA spike-in controls) were subjected to hybridization, rinsing, scanning and expression analysis using Agilent Whole Human Genome Microarrays (Human GE 4x44K V2 for analysis without RNA spike-in controls, SurePrint G3 Human GE 8x60K v2 for analysis with RNA spike-in controls). For data normalization, we used 75th percentile normalization for analysis without RNA spike-in controls, while for analysis with RNA spike-in controls, data were normalized using the median value for ERCC spike-in probes in each dataset [27]. Raw reads from these samples have been submitted to the Gene Expression Omnibus database of National Center for Biotechnology Information (accession no. GSE120291).

### Clustering analysis of microarray data

To conduct clustering analysis of microarray data, the distance matrix was calculated using Euclidean distance with the “ClassDiscovery” software package, version 3.3.7. Clustering analysis was performed with the hclust function in the “stats” package, version 3.2.4, using all genes or only genes on the disomic chromosomes [8].

### Statistical analyses

All statistical analyses were performed using R version 2.14.0 (http://www.r-project.org) software. Comparisons were made by Student’s t-test or Welch’s two-sample t-test. P < 0.05 was considered to be significant. Data and graphs are expressed as the mean ± standard deviation.

## Results

### Chromosome trisomies impair cellular proliferation and cause premature senescence in human fibroblasts

**Fig 1:**
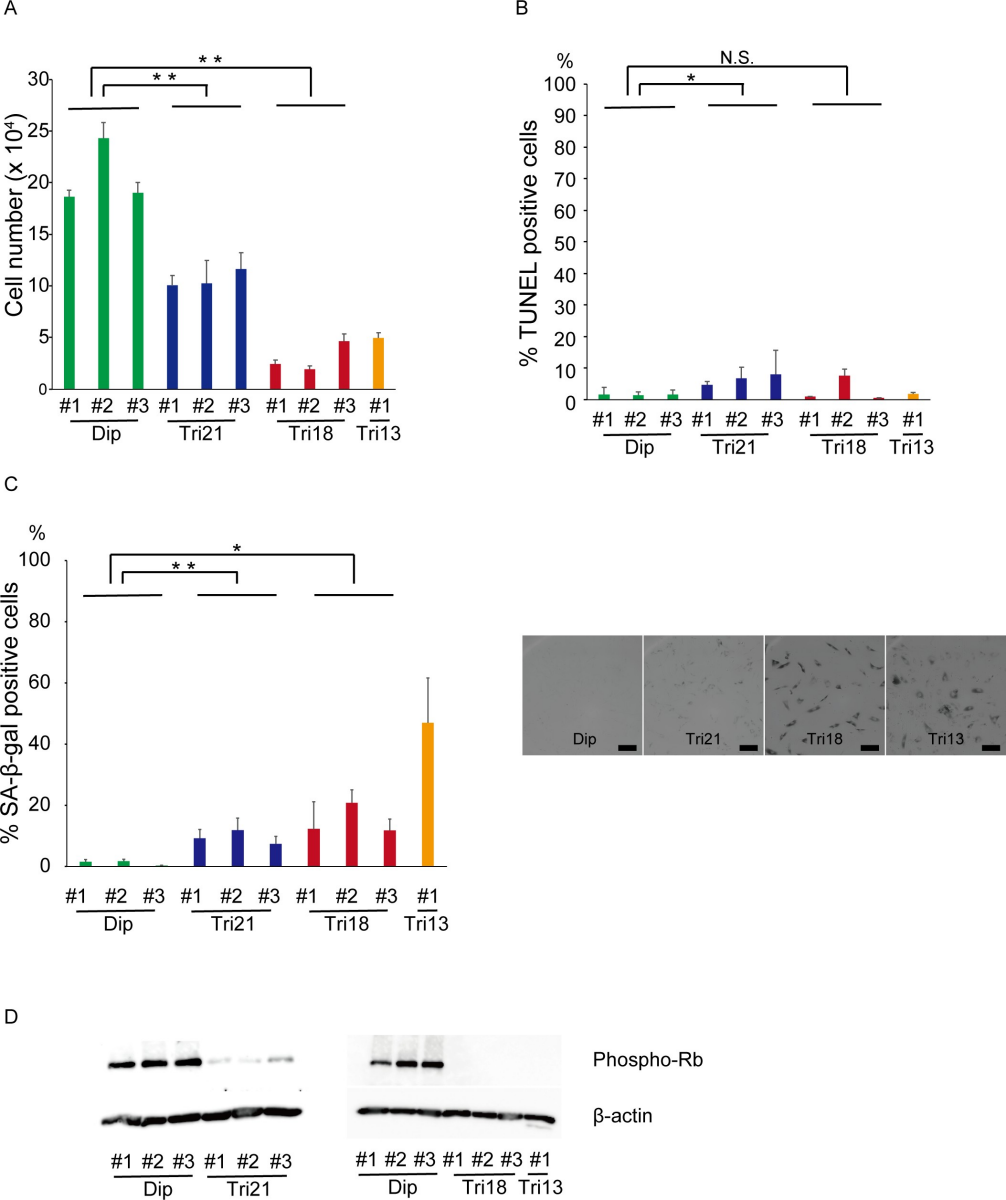
Trisomy fibroblasts showed severe proliferative impairment owing to premature senescence. (A) Absolute cell numbers three days after seeding 3×10^4^ cells (n = 3 per cell line). **P < 0.01. Dip, diploid; Tri, trisomy. (B) Percentage of TUNEL-positive cells (n = 3–7 per cell line). *P < 0.05. Dip, diploid; Tri, trisomy; N.S., not significant. (C) Percentage of SA-β-gal-positive cells (n = 3 per cell line). The right panel shows representative phase contrast images. Bar = 100 μm. **P < 0.01; *P < 0.05. Dip, diploid; Tri, trisomy. (D) Expression of phospho-Rb by western blotting analysis. Comparisons were made by Student’s t-test or Welch’s two-sample t-test.

In addition to chromosome-specific effects, aneuploidy has been reported to cause cellular stress in several species such as budding and fission yeast, and in mouse cells (aneuploidy-associated stresses) [7, 8, 14, 28, 29]. To examine whether these general effects can be extensively observed in naturally occurring human aneuploidies, we collected seven lines of human fibroblasts from autosomal trisomy syndromes (three lines from trisomy 21, three lines from trisomy 18, and one line from trisomy 13) and three lines from control donors, and analyzed the cell physiology of these primary fibroblasts. Trisomy 21 fibroblasts showed slower proliferation than controls, as previously reported [30, 31], and even more severe impairment was observed in the proliferation of cell lines with trisomy 18 and 13 (Fig 1A). TUNEL-positive cells were significantly increased in trisomy 21, but the difference was relatively small, and no similar change was seen in trisomy 18 and 13 cells, suggesting that apoptotic cell death was not the primary cause of reduced proliferation in trisomy cells (Fig 1B). Notably, all trisomy cell lines showed markedly increased populations of SA-β-gal-positive cells (Fig 1C). Suppression of phospho-Rb was also observed in trisomy cells (Fig 1D). These results are consistent with previous studies showing the association between aneuploidy and cellular senescence [32–35]. The severity of proliferation defects, the percentage of SA-β-gal-positive cells, and the expression changes in senescence-associated molecules were more obvious in trisomy 18 and 13 cells, but relatively mild in trisomy 21 cells. These results suggest that chromosome aneuploidy induces common physiological phenotypes, which are associated with the numbers of the genes located on the affected chromosomes.

### Cellular RNA and protein contents are increased in senescent trisomy fibroblasts

**Fig 2:**
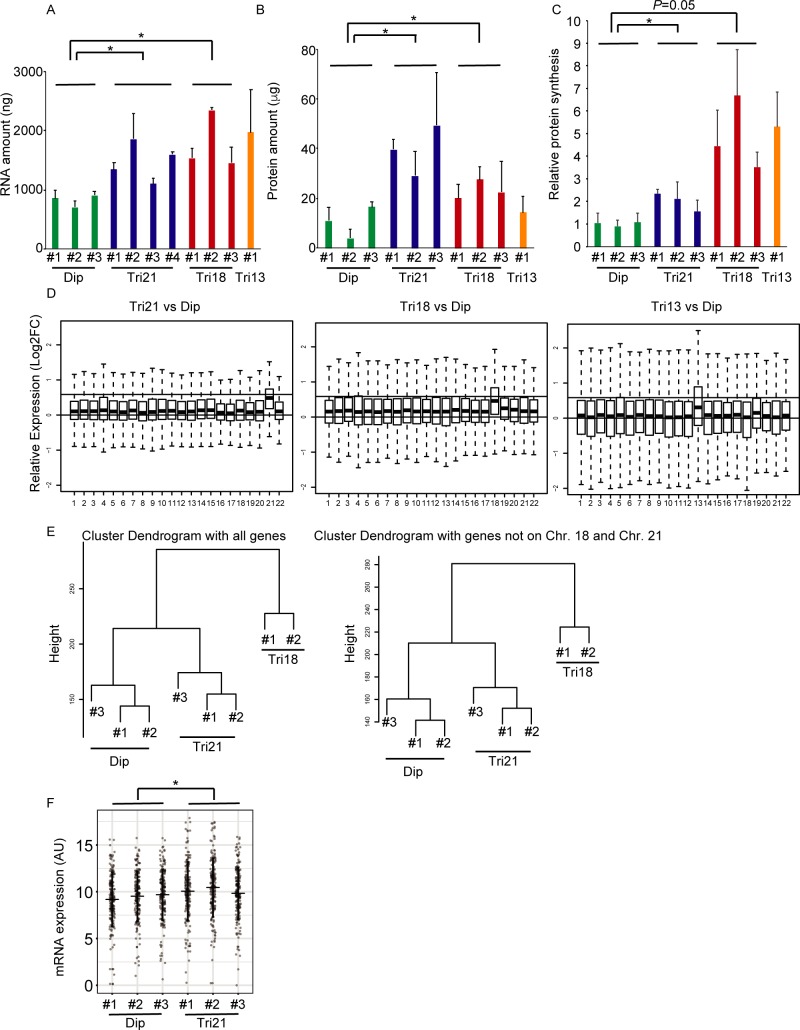
Senescent trisomy fibroblasts showed increased cellular RNA and protein contents. (A) Cellular RNA content in 1×10^5^ cells (n = 3–16 per cell line). *P < 0.05. Dip, diploid; Tri, trisomy. (B) Cellular protein content in 1×10^5^ cells (n = 3–6 per cell line). *P < 0.05. Dip, diploid; Tri, trisomy. (C) Relative protein synthesis ratio per cell, as assessed by the Click-iT AHA Alexa Fluor 488 Protein Synthesis HCS Assay Kit (n = 6–12 per cell line). *P < 0.05. Dip, diploid; Tri, trisomy. (D) Boxplots of relative log expression ratios for the genes on each chromosome using microarray data without the spike-in control. The left panel shows data for trisomy 21 fibroblasts compared with control fibroblasts. The middle panel shows data for trisomy 18 fibroblasts compared with the controls. The right panel shows data for trisomy 13 fibroblasts compared with the controls. The upper horizontal lines indicate a ratio of 1.5 (= 0.58 in log2 scale), while the lower horizontal lines indicate a ratio of 1.0 (= 0 in log2 scale). Dip, diploid; Tri, trisomy. (E) Hierarchical cluster analysis of microarray data with the spike-in control. The left panel shows a cluster dendrogram using all genes. The right panel shows a cluster dendrogram using only genes on the disomic chromosomes. Dip, diploid; Tri, trisomy. (F) Gene expression levels as assessed by NanoString’s nCounter analysis system. *P < 0.05. Dip, diploid; Tri, trisomy. Comparisons were made by Student’s t-test or Welch’s two-sample t-test.

Premature senescence is induced by various types of cellular stress mechanism [36, 37]. Trisomy fibroblasts undergoing accelerated premature senescence exhibited enlarged, flattened and irregular shapes, and these cells contained more than twice the amounts of total RNA as control cells (Fig 2A). In addition, cellular protein amounts and synthesis ratios were markedly increased in trisomic cells, compared with those in diploid fibroblasts (Fig 2B and 2C). Protein synthesis in trisomy 18 cells showed a consistently increasing trend, although the increase was marginally significant, possibly because of the variability among these trisomy cells. To explore global gene expression, microarray analysis was performed. The expression levels of genes on trisomic chromosomes (chromosome 13, 18 or 21) were increased approximately 1.5-fold on average compared with diploid cells, as expected (Fig 2D). Intriguingly, small but distinct levels of global transcriptional upregulation were observed even for genes on non-trisomic (disomic) chromosomes.

In general, transcriptional analysis is conducted under the assumption that equivalent amounts of RNA are produced per cell. However, it has been reported that introducing similar amounts of total RNA from cell extracts into the experimental platform and applying standard normalization methods may mask the widespread increase of transcription (transcriptional amplification) and lead to an erroneous interpretation when the total level of mRNA is different between the cell lines under comparison [27, 38]. To perform a more robust transcriptional analysis, we added extrinsic spiked-in RNA standards to total RNA extracts in proportion to the number of cells, and the microarray datasets were normalized using these spiked-in controls. As reported in S2 Fig, we obtained similar results after normalization with the spiked-in controls. Hierarchical cluster analysis of the microarray data indicated relative similarity in gene expression among cells of the same karyotype, and differences between diploid, trisomy 21 and trisomy 18. Notably, the differences were conserved when only genes encoded on chromosomes other than 18 and 21 were analyzed, suggesting that chromosome trisomy affects the global gene expression pattern (Fig 2E). This transcriptional amplification was confirmed by NanoString’s nCounter analysis system, in which multiplexed gene expression levels were directly detected without enzymatic reactions [39]. mRNA levels/cell were quantified for 234 genes encoded on various chromosomes other than chromosome 21. The results showed that transcripts from 173 genes (73.9%) were expressed at reliable levels in all three diploid cell lines, and mean gene expression levels were higher in senescent trisomy 21 fibroblasts, with an average 2.2-fold increase in expression (Fig 2F). These results indicate that gene transcription and protein synthesis are globally amplified in senescent trisomy fibroblasts.

### Energy metabolism is disturbed in senescent trisomy fibroblasts

**Fig 3:**
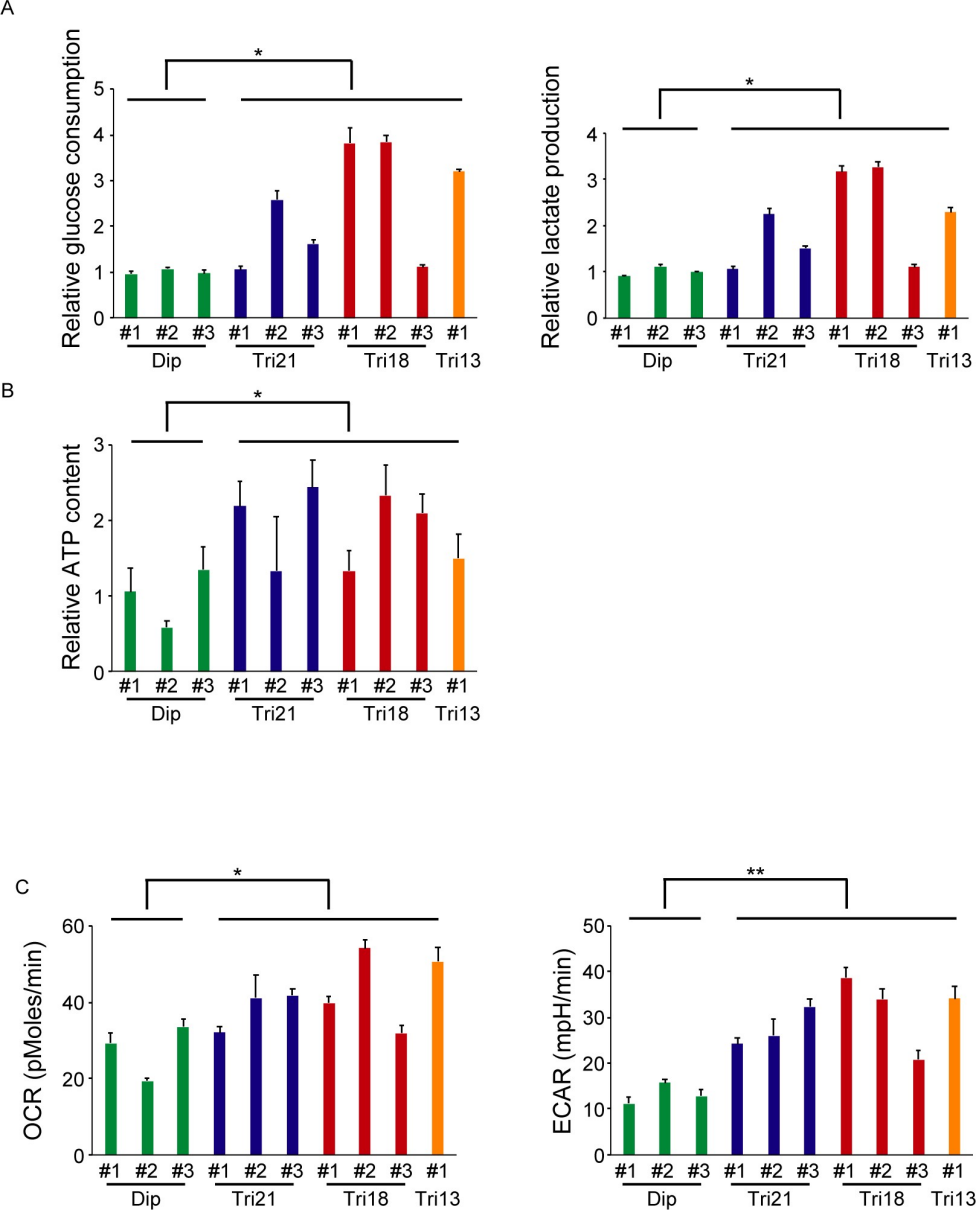
Senescent trisomy fibroblasts showed increased glucose consumption and increased lactate production, suggesting disturbed energy metabolism. (A) The left panel shows relative glucose consumption by 1×10^5^ cells over three days, as assessed by decreased glucose content in the culture medium. The right panel shows relative lactate production by 1×10^5^ cells over three days, as assessed by increased lactate content in the culture medium (n = 3 per cell line). *P < 0.05. Dip, diploid; Tri, trisomy. (B) Relative cellular ATP content, as assessed by an intracellular ATP determination kit (n = 4 per cell line). *P < 0.05. Dip, diploid; Tri, trisomy. (C) The left panel shows oxygen consumption rate (OCR), as assessed by an XF96 Extracellular Flux Analyzer. *P < 0.05. The right panel shows extracellular acidification rate (ECAR), as assessed by an XF96 Extracellular Flux Analyzer (n = 3–6 per cell line). **P < 0.01. Dip, diploid; Tri, trisomy. Comparisons were made by Student’s t-test or Welch’s two-sample t-test.

Amplified global gene transcription and increased protein synthesis might alter energy metabolism in senescent trisomy fibroblasts. Indeed, extracellular glucose uptake measurements revealed markedly increased glucose consumption by senescent trisomy cells. In agreement with increased glucose consumption, these trisomy fibroblasts showed increased lactate production (Fig 3A). Intracellular ATP levels were markedly increased in trisomy fibroblasts, and inhibition of ATP production by treatment with oligomycin resulted in a significantly greater reduction in ATP concentration in trisomy 21 cells, suggesting their accelerated consumption of ATP (Fig 3B and S3A Fig). Measurements of the oxygen consumption rate (OCR) and extracellular acidification rate (ECAR) on same population of cells showed that they exhibited accelerated utilization of glycolysis and oxidative phosphorylation (Fig 3C). Furthermore, inhibition of RNA or protein synthesis by treatment with actinomycin D or cycloheximide showed a pronounced decrease of OCR values in trisomy 21 fibroblasts (albeit not statistically significant in data from the RNA synthesis inhibition assay) (S3B Fig). These results suggest that global gene transcriptional amplification and accelerated protein synthesis cause disturbed cellular energy metabolism in senescent trisomy fibroblasts.

### Chromosome aneuploidy causes oxidative stress and mitochondrial damage

**Fig 4:**
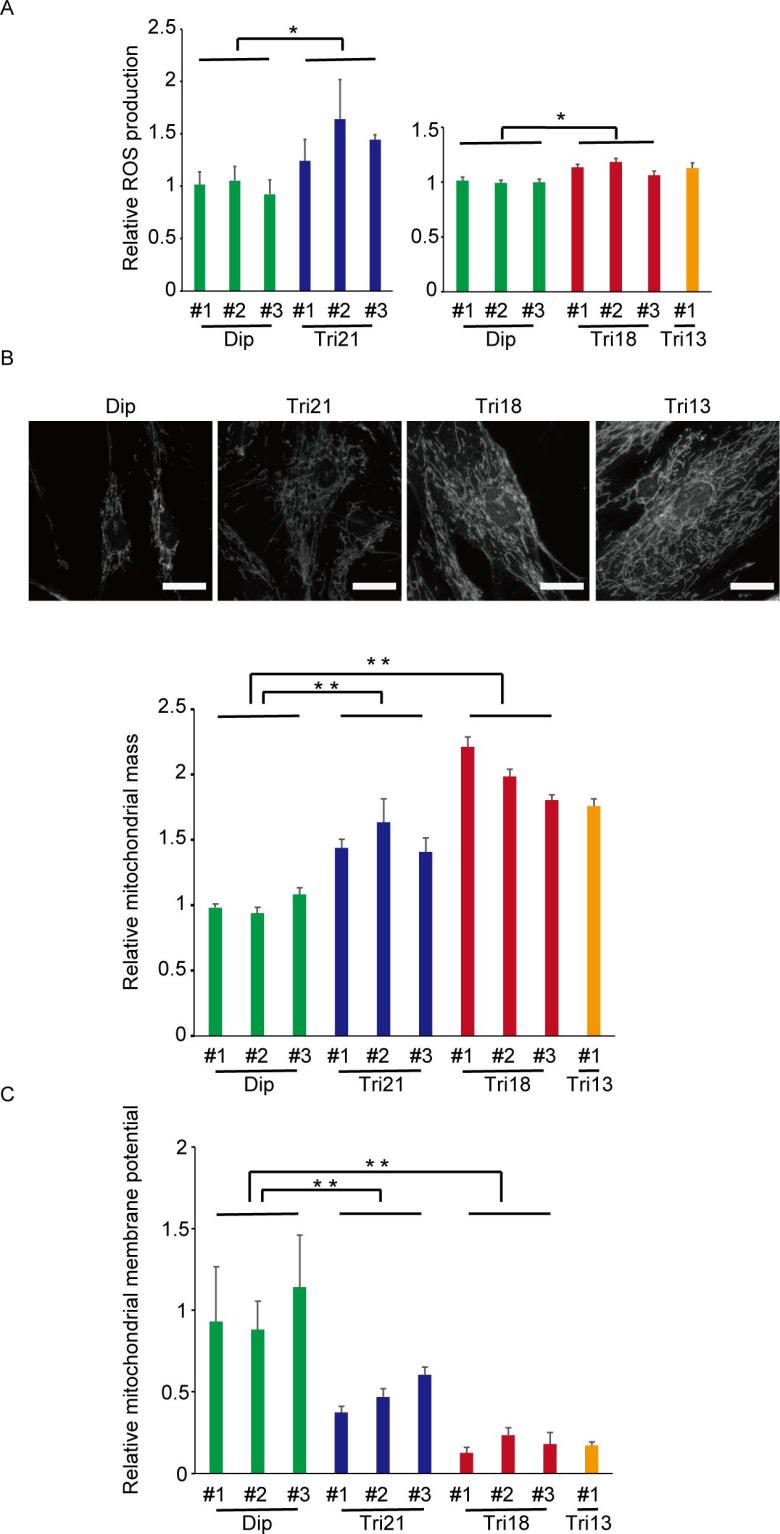
Trisomy fibroblasts showed increased oxidative stress and accumulation of damaged mitochondria. (A) Relative ROS production as expressed by relative MitoSOX/MitoTracker ratio (n = 5–6 per cell line). *P < 0.05. Dip, diploid; Tri, trisomy. (B) Relative mitochondrial mass per cell as assessed by average TOM20-positive area per cell. The upper panels show representative phase contrast images in which mitochondria were stained with MitoTracker. Bar = 50 μm. The lower panel shows corresponding quantification (n = 3–8 per cell line). **P < 0.01. Dip, diploid; Tri, trisomy. (C) Relative mitochondrial membrane potential per cell (n = 5–10 per cell line). **P < 0.01. Dip, diploid; Tri, trisomy. Comparisons were made by Student’s t-test.

Activated intracellular energy metabolism can lead to constitutive generation of ROS [40]. Analysis of ROS production using an oxidant-sensitive fluorogenic probe revealed an excessive production of ROS in all three trisomy lines (Fig 4A). In addition, the total cross-sectional areas of mitochondria per cell were significantly increased in trisomy fibroblasts compared with disomic controls, and mitochondrial membrane potential was severely decreased in all the trisomy lines (Fig 4B and 4C) [19]. Notably, the alterations in both mitochondrial mass and membrane potential observed in trisomy 18 and 13 were even more severe than those in trisomy 21. These results suggest that autosomal chromosome trisomy exerts oxidative stress and mitochondrial damage in fibroblasts.

### A chemical chaperone reduces the accumulation of protein aggregates in trisomy fibroblasts

**Fig 5:**
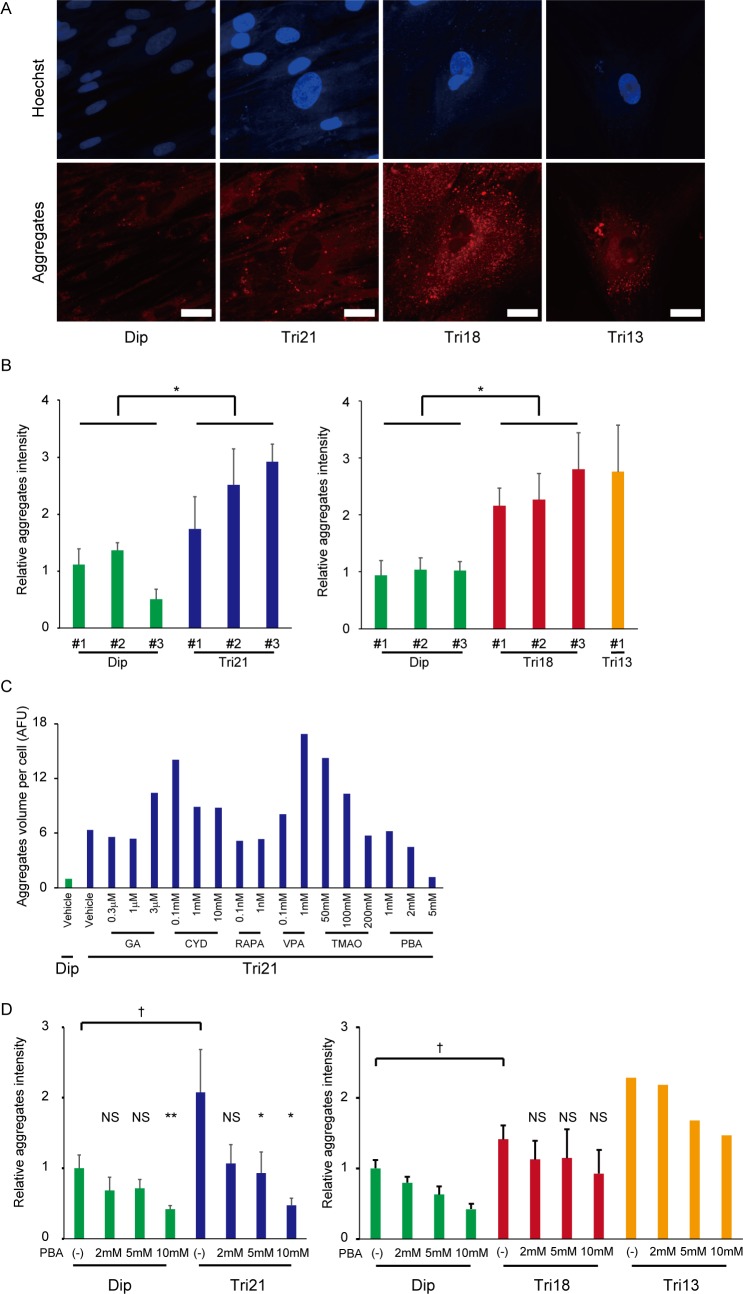
Accumulation of aggregated proteins and effect of the chemical chaperone, 4-PBA in trisomy fibroblasts. (A) Accumulation of aggregated proteins, as detected by the PROTEOSTAT Aggresome Detection kit. Bar = 50 μm. Dip, diploid; Tri, trisomy. (B) Corresponding quantification of aggregated protein accumulation (n = 5–6 per cell line). *P < 0.05. Dip, diploid; Tri, trisomy. The left panel shows the result for the comparison between diploid and trisomy 21 cell lines. The right panel shows the result for the comparison between diploid and trisomy 18 and 13 cell lines. (C) Quantification of aggregated protein accumulation after culture in the presence of six chemical compounds: GA, geldanamycin; CYD, 2-hydroxypropyl-β-cyclodextrin; RAPA, rapamycin; VPA, valproic acid sodium salt; TMAO, trimethylamine N-oxide; PBA, sodium phenylbutyrate. Dip, diploid; Tri, trisomy. (D) Effects of sodium phenylbutyrate on aggregated protein accumulation (mean of three cell lines for Dip, Tri21, and Tri18 fibroblasts; n = 3 per cell line). Comparison of trisomy samples without PBA with diploid samples without PBA, ^†^P < 0.05. Comparison of samples with PBA with samples without PBA, *P < 0.05, **P < 0.01. PBA, sodium phenylbutyrate; Dip, diploid; Tri, trisomy; N.S., not significant.

Oxidative stress has an important role in protein homeostasis. A moderate oxidant concentration increases proteasomal degradation, whereas persistent and higher oxidant levels lead to proteolytic inhibition and the accumulation of misfolded protein [41–43]. To examine the intracellular effects of dysregulated protein homeostasis in trisomy cells, aggregated protein was detected using an aggregation-sensitive molecular rotor dye [44]. Red fluorescent foci were localized largely in the cytoplasm, and trisomy cells showed obviously greater aggregation (Fig 5A). Moreover, signal intensities were significantly higher in trisomy 21, 18 and 13 cells, suggesting that the severity of trisomy-induced cellular stress affects the level of aggregate formation (Fig 5B).

Since the accumulation of misfolded proteins is associated with several human diseases, this process is considered to be a potential therapeutic target [45, 46]. To examine whether preventing the formation and/or removal of protein aggregates can ameliorate the various pathological phenotypes observed in trisomy syndromes, fibroblasts were cultured with several chemical compounds, and aggregate intensities were then evaluated. Protein aggregation levels were elevated in trisomy 21 fibroblasts, and most of the compounds tested were not only ineffective, but even exacerbated the production of aggregates (Fig 5C). However, sodium 4-PBA, a potent chemical chaperone, decreased aggregate formation without showing any harmful effects. This effect was further tested in the other trisomy fibroblast lines, and administration of 4-PBA exhibited remarkable aggregate-reducing effects in a concentration-dependent manner (Fig 5D, S4 Fig).

### Premature senescence can be rescued by 4-PBA in trisomy 21 iPSC-derived secondary fibroblasts

Since several human diseases are caused by defects in protein folding, reduction or removal of protein aggregates may ameliorate the pathological phenotype. Premature senescence is thought to be triggered by various types of stimuli, and then locked into irreversible growth arrest by epigenetic alteration [47]. To investigate whether pretreatment with 4-PBA delayed or prevented the induction of trisomy-associated premature senescence, we used trisomy 21 fibroblast-derived human iPSCs (Tri21 iPSCs) and secondary fibroblasts. To exclude effects of the genetic background, we used a corrected disomy 21 iPSC line (cDi21 iPSC) as a control, in which a single copy of chromosome 21 was removed from a Tri21 iPSC line using chromosome editing technology [22]. These two iPSC lines perfectly share the same genetic background, enabling an ideal comparison of cellular phenotypes.

**Fig 6:**
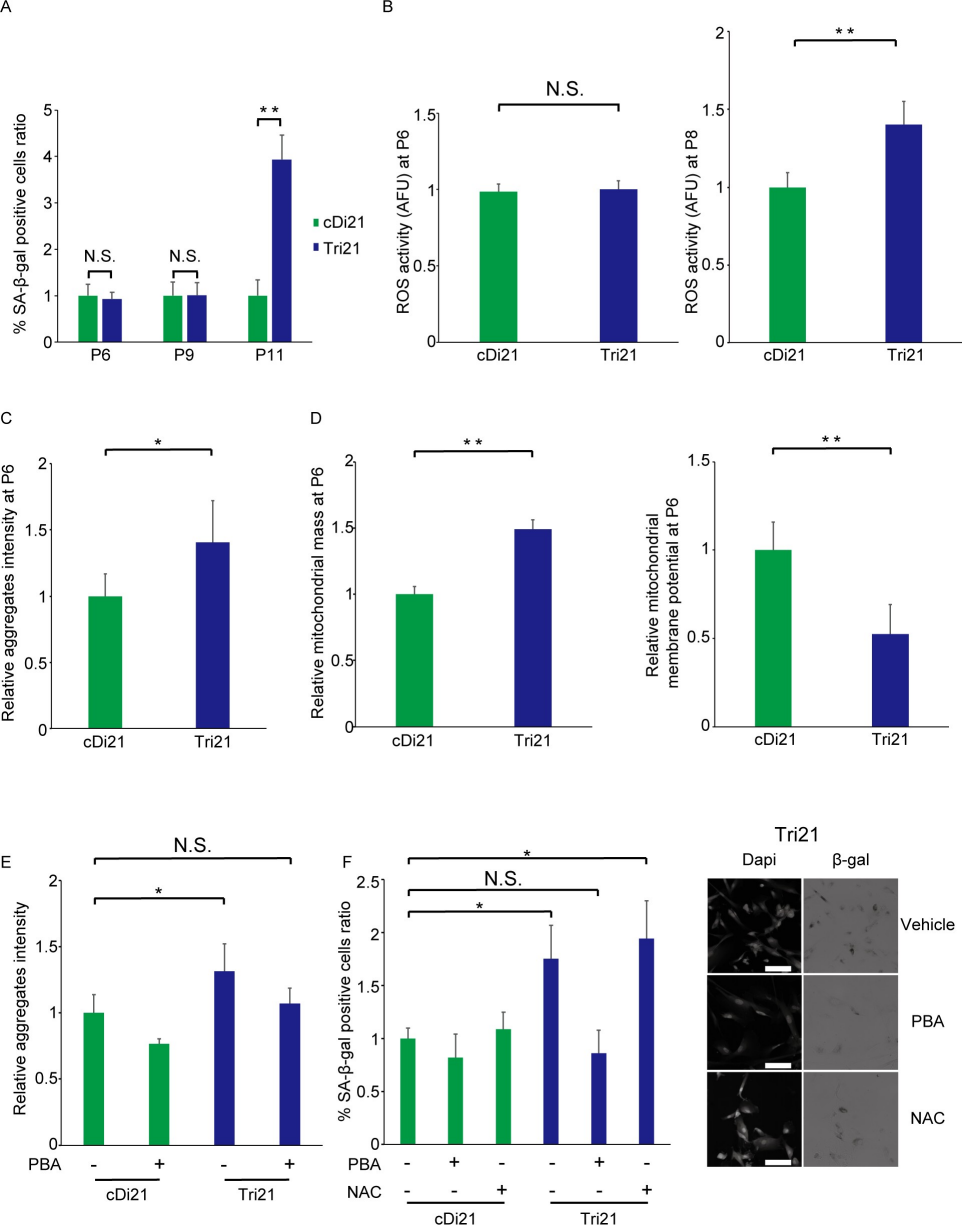
Rescue of trisomy-induced premature senescence by 4-PBA treatment in trisomy 21 iPSC-derived secondary fibroblasts. (A) Percentage of SA-β-gal-positive cells (n = 3 per cell line). **P < 0.01. cDi21, corrected disomy 21 iPSC-derived secondary fibroblasts; Tri21, trisomy 21 iPSC-derived secondary fibroblasts; N.S., not significant. (B) Relative ROS production as expressed by relative MitoSOX/MitoTracker ratio at passage 6 (left) and passage 8 (right) (n = 6 per cell line). **P < 0.01. cDi21, corrected disomy 21 iPSC-derived secondary fibroblasts; Tri21, trisomy 21 iPSC-derived secondary fibroblasts; N.S., not significant. (C) Accumulation of aggregated proteins, as detected by the PROTEOSTAT Aggresome Detection kit (n = 6 per cell line). *P < 0.05. cDi21, corrected disomy 21 iPSC-derived secondary fibroblasts; Tri21, trisomy 21 iPSC-derived secondary fibroblasts. (D) The left panel shows relative mitochondrial mass per cell as assessed by the mean MitoTracker Red CMXRos-positive area per cell. The right panel shows relative mitochondrial membrane potential per cell (n = 6 per cell line). **P < 0.01. cDi21, corrected disomy 21 iPSC-derived secondary fibroblasts; Tri21, trisomy 21 iPSC-derived secondary fibroblasts. (E) Effects of 4-PBA on aggregated protein accumulation (n = 5 per cell line). *P < 0.05. PBA, sodium phenylbutyrate; cDi21, corrected disomy 21 iPSC-derived secondary fibroblasts; Tri21, trisomy 21 iPSC-derived secondary fibroblasts; N.S., not significant. (F) Effects of 4-PBA (2 mM) or NAC (2.5 mM) on cellular senescence. The right panel shows representative phase contrast images. Bar = 100 μm. The left panel shows corresponding quantification (n = 3 per cell line). *P < 0.05. PBA, sodium phenylbutyrate; NAC, N-acetyl-L-cysteine; cDi21, corrected disomy 21 iPSC-derived secondary fibroblasts; Tri21, trisomy 21 iPSC-derived secondary fibroblasts; N.S., not significant.

Both iPSC lines showed only sparse (less than 0.2%) SA-β-gal-positive cells, indicating that epigenetic modification and cellular senescence were reset in the process of reprogramming (S5 Fig). These cell lines were then differentiated into secondary fibroblasts, to evaluate the development of senescence [23]. In the early differentiation stage at passage 6, there were no differences in the ratio of SA-β-gal-positive cells between Tri21 and cDi21 cell lines, indicating that the cells had not entered the senesced state yet (Fig 6A). In the differentiation process of pluripotent stem cells, the cellular metabolic system is known to dramatically change from glycolysis to oxidative phosphorylation, which is accompanied by increased oxygen consumption and reactive oxygen species levels. Reflecting this change, an elevation of ROS in trisomy 21 cells was not evident at P6, but became apparent at P8 (Fig 6B). Even at this early point, the accumulation of protein aggregates was significantly higher in Tri21 cells compared with cDi21 cells at passage 6 (Fig 6C). In addition, increased mitochondrial mass and reduced mitochondrial membrane potential indicated that trisomy-induced stress had already affected the cells by this passage number (Fig 6D). At an advanced differentiation stage (P11), premature senescence as a cellular phenotype became evident in Tri21-derived secondary fibroblast cells (Fig 6A).

On the basis of our finding that 4-PBA effectively eliminates protein aggregates in fibroblasts, this compound was applied to cDi21/Tri21-derived secondary fibroblasts from the point of passage 6. Protein aggregates levels were significantly elevated in untreated Tri21 cells, but dramatically decreased to the same levels in cDi21 cells when treated with 4-PBA (Fig 6E). Notably, the ratio of SA-β-gal-positive cells among Tri21 secondary fibroblasts, which was significantly increased at this stage, was successfully suppressed to the same level as that in cDi21 cells (Fig 6F). In contrast, no significant decrease of the SA-β-gal-positive cell ratio was observed in NAC-treated Tri21 secondary fibroblasts, suggesting that prevention of protein aggregation may be a more potent therapeutic target compared with the oxidative stress pathway. These results suggest that 4-PBA may be useful for the prevention of premature senescence caused by the trisomy-induced stress.

## Discussion

Cellular responses to karyotypic alteration have been intensively investigated in various experimental models such as yeast, mice and humans [7, 10, 14, 48]. These studies demonstrated that aneuploidies with different sets of chromosomes exert common proteotoxic stress and cause transcriptional and/or posttranscriptional alteration, leading to defects in multiple biological processes [8, 29, 49]. To extend these important insights, we collected human fibroblasts from patients with autosomal trisomy syndromes (Patau syndrome, trisomy 13; Edwards syndrome, trisomy 18; Down syndrome, trisomy 21) and examined the pathophysiological mechanisms shared by these syndromes. Trisomy fibroblasts showed common proliferation defects and accelerated premature senescence, consistent with earlier results in yeast [15]. These cells were large, flattened, and contained increased amounts of RNA and proteins. Interestingly, this biological response was accompanied by the global upregulation of non-trisomic gene expression, which could be uncovered using extrinsic spike-in standards for normalization. Transcriptional amplification, an increase in global levels of mRNAs, has been implicated in tumors, heart failure and aging [50–52]. Several transcriptional regulators that bind to promoters or enhancers, such as c-Myc, have been thought to play a central role in these phenomena. However, the gene sets located on chromosomes 21, 18 and 13 are different, and therefore, there are no common dosage alterations in transcriptional regulators between these trisomies. In senescing trisomy fibroblasts, each chromosome-specific dosage-dependent expression of trisomic genes is a first step, and increased expression of several hundreds of genes may then stimulate larger-scale transcriptional network responses, leading to the activation of various transcription regulating proteins, rather than responses being mediated by the specific actions of a few regulators such as c-Myc. At present, it is unclear whether this altered gene expression is a primary cause or simply an effect of premature senescence. However, we have observed similar upregulation of RNA/protein synthesis in other differentiated cells that do not exhibit premature senescence, suggesting that upregulated expression may be involved in the pathological mechanism in trisomy fibroblasts, and not be a mere consequence of senescence (unpublished data).

Our microarray data demonstrated that the extra chromosome in trisomy 13 cells was not expressed as highly as the others. The karyotype analysis confirmed that all primary trisomy fibroblasts retained their original trisomy karyotypes. It was possible that minor mosaicism, which was not revealed by the karyotype analyses, could explain the finding. However, another possible explanation was that, while chromosomal dosage compensation has only been established for sex chromosomes, previous studies have also found that dosage compensation of aneuploidy can occur for autosomes [53, 54]. The suggested mechanisms include feedback regulation for each single gene and an unknown protein that can buffer the expression of the genes on extra chromosomes, an example of which is the POF protein for the fourth chromosome in *Drosophila melanogaster* [53, 54]. Further study is needed to investigate the gene expression levels on the extra chromosome and possible mechanism of the modified expression on the extra chromosomes using human trisomy 13 cells.

Increased energy demand to synthesize excessive amounts of RNA and protein should impose a metabolic burden on trisomy cells, followed by mitochondrial dysfunction and ROS production that was similarly observed in aneuploid yeast cells [12, 13]. Consistent with a previous study, ROS levels were significantly increased in the trisomy 21 cells [55]. Although ROS levels in trisomy 13 cells could not be statistically evaluated due to the lack of cell lines, ROS production was similarly elevated in trisomy 18 cells, suggesting the existence of a trisomy-induced stress effect.

Aneuploidy-associated stress is closely related to the total dosage of gene expression, and the numbers of trisomic genes are generally thought to affect the severity of the stress. Therefore, trisomies of chromosomes 13 and 18, which contain ~500 and ~400 genes, respectively, can exert more detrimental effects than that of chromosome 21, which harbors ~300 genes. This non-specific, but dose-dependent, stress action makes it even more difficult to understand the pathological mechanisms of trisomies. Nevertheless, its dose-dependency might explain, at least partly, why only fetuses with trisomies of chromosomes 21, 18, and 13, which have the least numbers of genes among all autosomal chromosomes, can survive to birth, and other trisomies are embryonic lethal, and why patients with trisomy 21 can live until 60 years of age on average.

Levels of ROS in fibroblasts with trisomy 18 were not statistically different from those in fibroblasts with trisomy 21. Such fluctuation was similarly observed in the cellular protein amount. Because any phenotype observed in trisomy cells results from the synergistic combination of the gene dosage effects and aneuploidy-associated stress, such discrepancy may result from the dosage effect caused by specific genes on trisomic chromosome. In this study, the aneuploidy-associated stress was simply defined as the common phenomena shared by three trisomies, regardless of those with different severities. To test the hypothesis, further experiments using trisomy 13 and 18 iPSC-derived secondary fibroblasts will be required as the next step.

Excessive ROS cause structural changes and misfolding of proteins, giving rise to the accumulation of insoluble and nondegradable cross-linked aggregates [56]. A proportional relationship exists between the amount of protein aggregates and the progression of aging, and more directly, the incubation of cells with artificial or isolated protein aggregates inhibits proteasome activity, causing some features of premature senescence [57, 58]. We therefore focused on the toxicity caused by these protein aggregates, and explored chemical compounds that might effectively reduce trisomy-associated protein aggregation.

A chemical chaperone, 4-PBA has hydrophobic regions that interact with exposed hydrophobic segments of an unfolded protein. This interaction promotes correct folding and structure stabilization of proteins under normal conditions. Under stress conditions, chaperones can act early in the aggregation process to maintain the solubility of disease proteins and prevent undesirable degradation and/or aggregation [59]. In our experiments, 4-PBA rapidly attenuated the levels of protein aggregates both in trisomy fibroblasts and in secondary fibroblasts differentiated from Tri21 iPSCs. Intriguingly, administration of 4-PBA efficiently reverted the percentages of SA-β-gal-positive cells in differentiated Tri21 secondary fibroblasts, indicating that aneuploidy-associated stress might be a potential therapeutic target for the pathogenic features of Down syndrome.

Although the toxic effects of protein aggregates on cell viability are evident, whether the suppressive action of 4-PBA on premature senescence was directly attributable to decreased protein aggregation or to some other unique mechanism is unclear. In addition to its primary effect as a chaperone, 4-PBA also acts as a histone deacetylase inhibitor and regulates the transcription of many genes involved in the protein quality control system. Previous studies have reported that 4-PBA treatment induces dramatic alterations in the gene expression pattern, including upregulation of genes specifically involved in molecular chaperoning, such as heat shock proteins, and downregulation of genes involved in metabolic pathways [60, 61]. Overall generalized changes in epigenetic regulation by 4-PBA may induce fine-tuning of transcriptional regulation, leading to an advantageous effect in this regard.

The effects of 4-PBA in inhibiting aggregate formation in cultured cells and its therapeutic potency in disease models have been proven in various types of experiments [62, 63]. 4-PBA is a blood–brain barrier-permeable and orally bioavailable drug, and importantly, has already been approved by the US Food and Drug Administration for the treatment of urea cycle disorders. Further study will be required to elucidate to what extent aneuploidy-associated stress is mechanistically involved in the various complications of Down syndrome, including neural dysfunction, and whether 4-PBA is effective for each phenotype.

## Supporting information

S1 Fig Karyotype analyses of primary trisomy fibroblasts. (A) Karyotype analysis of primary trisomy 21 fibroblasts. (B) Karyotype analysis of primary trisomy 18 fibroblasts. (C) Karyotype analysis of primary trisomy 13 fibroblasts.
(TIF)

S2 Fig Relative log expression ratios for genes on each chromosome using microarray data with the spike-in control. The left panel shows boxplots of the relative log expression ratios of genes on each chromosome in trisomy 21 fibroblasts as compared with control fibroblasts. The right panel shows similar data for trisomy 18 fibroblasts compared with controls. The upper horizontal lines indicate a ratio of 1.5 (= 0.58 in log2 scale), while the lower horizontal lines indicate a ratio of 1.0 (= 0 in log2 scale). Dip, diploid; Tri, trisomy.
(TIF)

S3 Fig Senescent trisomy fibroblasts have a disturbed energy metabolism. (A) Decreases in cellular ATP levels following inhibition of ATP production by oligomycin (2 μM; n = 3 per cell type). *P < 0.05. Dip, diploid; Tri, trisomy. (B) Left panel, decreases in the oxygen consumption rate (OCR) following inhibition of RNA synthesis using actinomycin D (1 μg/ml). Right panel, decreases in the OCR following inhibition of protein synthesis using cycloheximide (0.5 μg/ml; n = 3 per cell line). *P < 0.05. Dip, diploid; Tri, trisomy; N.S., not significant. Comparisons were made by the Student’s t-test or Welch’s two-sample t-test.
(TIF)

S4 Fig Effects of sodium phenylbutyrate on aggregated protein accumulation. Data of three diploid and three trisomy 21 fibroblast cell lines are shown (n = 3 per cell line; original data in Fig 5D). *P < 0.05. PBA, sodium phenylbutyrate; Dip, diploid; Tri, trisomy; N.S., not significant.
(TIF)

S5 Fig SA-β-gal expression in iPSCs. Percentages of SA-β-gal positive cells were calculated for undifferentiated iPSC lines (n = 4 per cell line). cDi21, corrected disomy 21 iPSCs; Tri21, trisomy21 iPSCs; N.S., not significant.
(TIF)

S1 Table Characteristics of samples in the present study. Information on sex and age at sample collection for each patient is shown.
(DOCX)
