# Supplementary figures and images for: Elimination of protein aggregates prevents premature senescence in human trisomy 21 fibroblasts
Source: PLoS One. 2019 Jul 29;14(7):e0219592. doi: 10.1371/journal.pone.0219592 (PMC6663065; doi:10.1371/journal.pone.0219592)

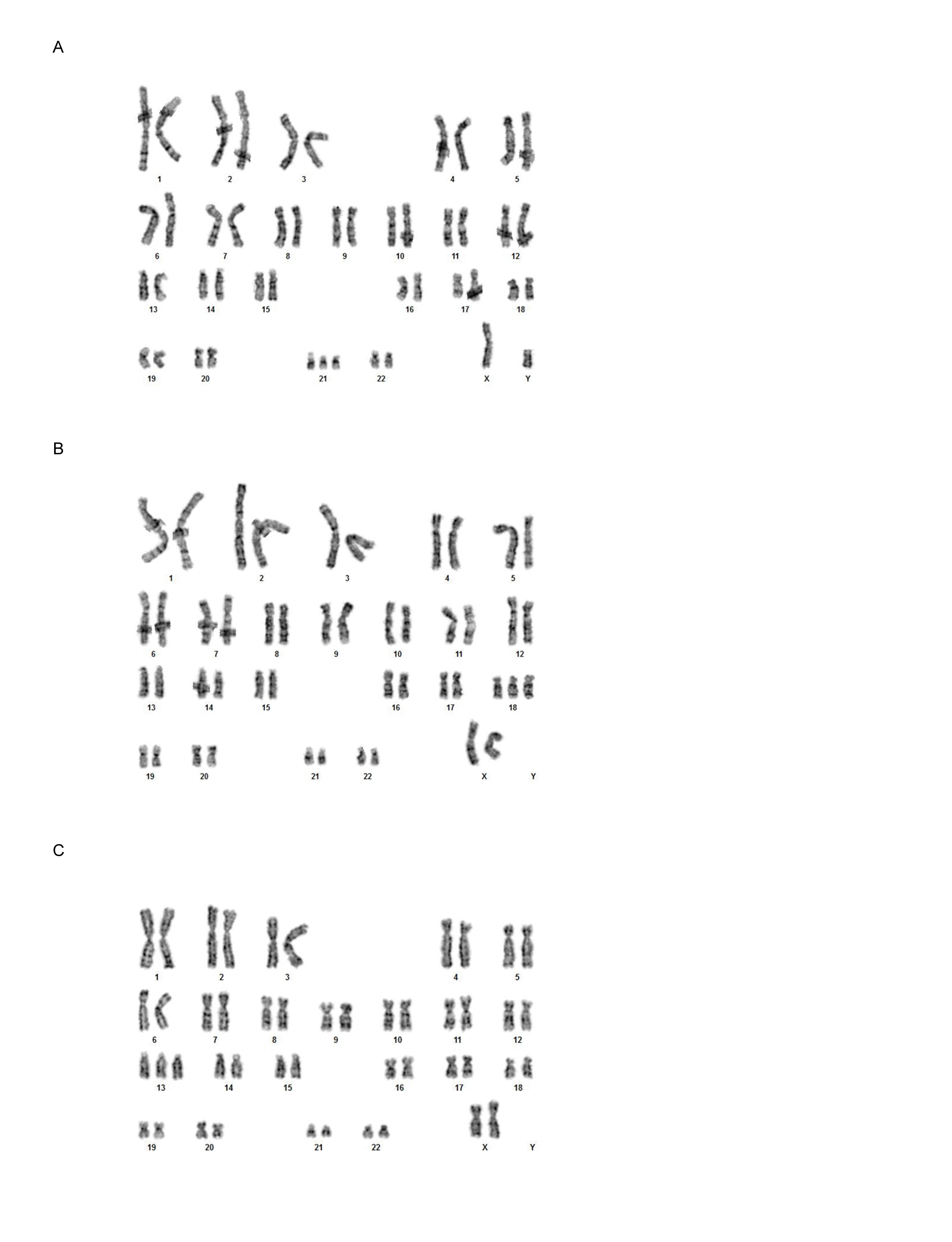

Supplement: S1 Fig — (A) Karyotype analysis of primary trisomy 21 fibroblasts. (B) Karyotype analysis of primary trisomy 18 fibroblasts. (C) Karyotype analysis of primary trisomy 13 fibroblasts. (TIF) [file pone.0219592.s001.tif]

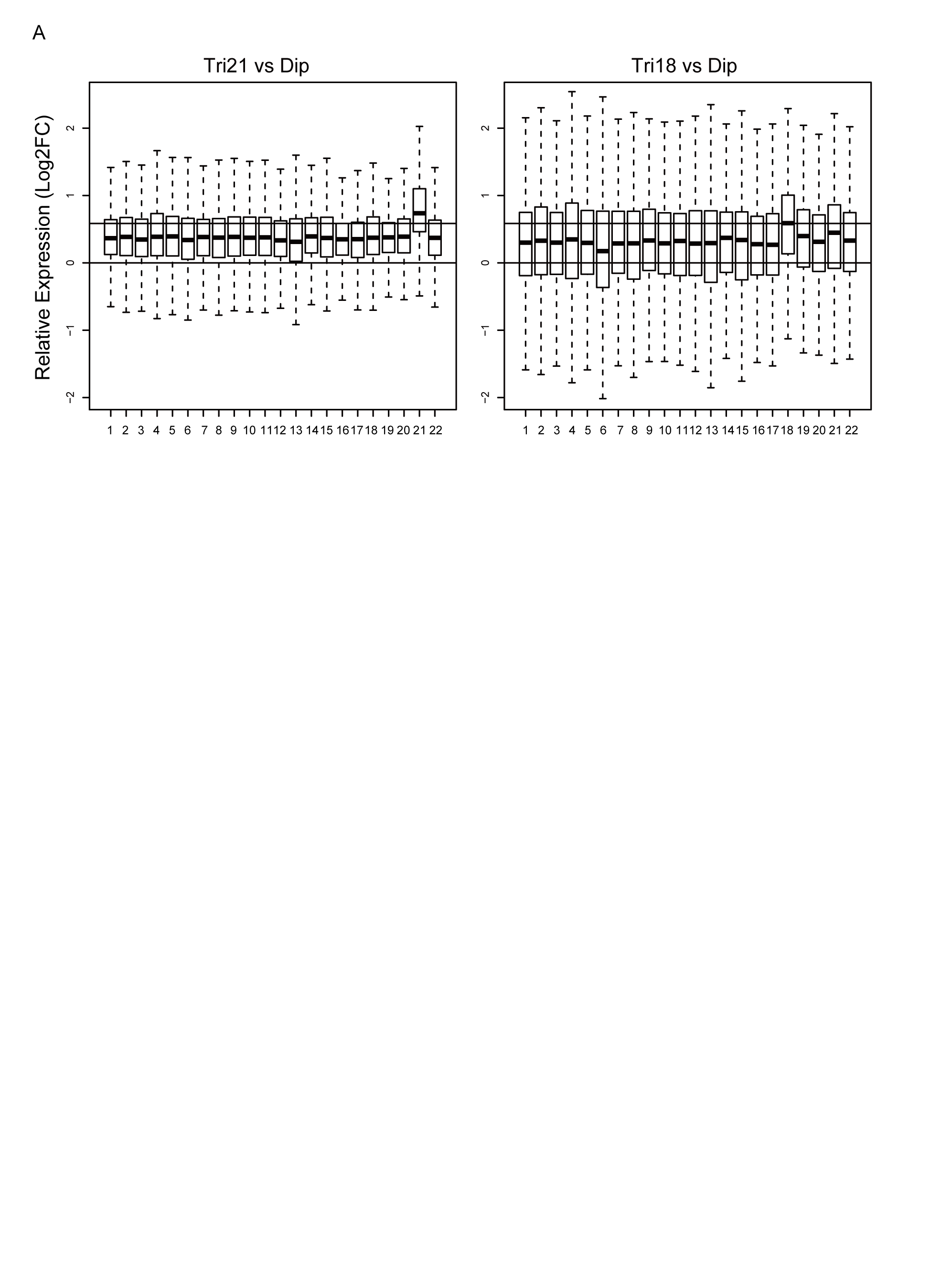

Supplement: S2 Fig — The left panel shows boxplots of the relative log expression ratios of genes on each chromosome in trisomy 21 fibroblasts as compared with control fibroblasts. The right panel shows similar data for trisomy 18 fibroblasts compared with controls. The upper horizontal lines indicate a ratio of 1.5 (= 0.58 in log2 scale), while the lower horizontal lines indicate a ratio of 1.0 (= 0 in log2 scale). Dip, diploid; Tri, trisomy. (TIF) [file pone.0219592.s002.tif]

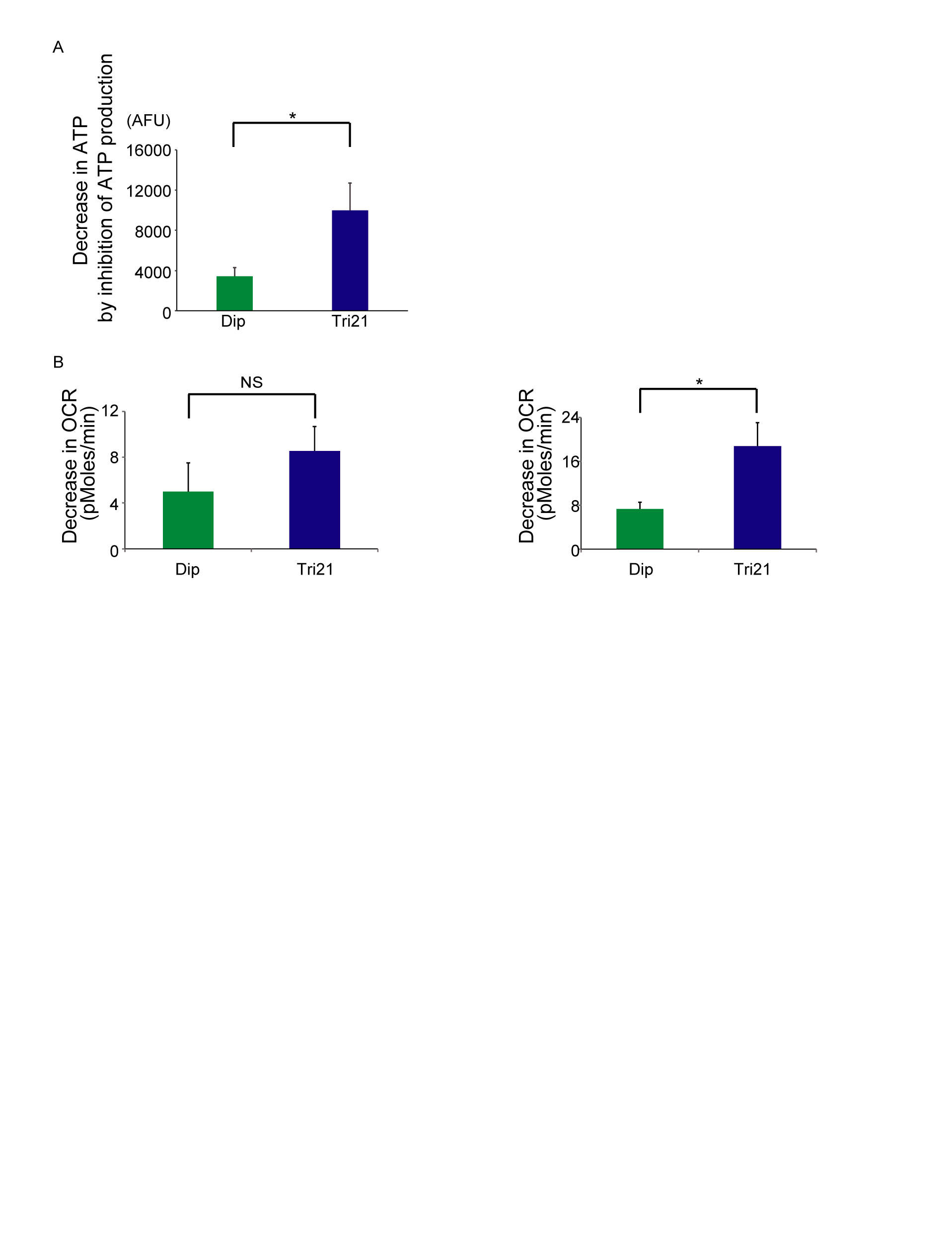

Supplement: S3 Fig — (A) Decreases in cellular ATP levels following inhibition of ATP production by oligomycin (2 μM; n = 3 per cell type). *P < 0.05. Dip, diploid; Tri, trisomy. (B) Left panel, decreases in the oxygen consumption rate (OCR) following inhibition of RNA synthesis using actinomycin D (1 μg/ml). Right panel, decreases in the OCR following inhibition of protein synthesis using cycloheximide (0.5 μg/ml; n = 3 per cell line). *P < 0.05. Dip, diploid; Tri, trisomy; N.S., not significant. Comparisons were made by the Student’s t-test or Welch’s two-sample t-test. (TIF) [file pone.0219592.s003.tif]

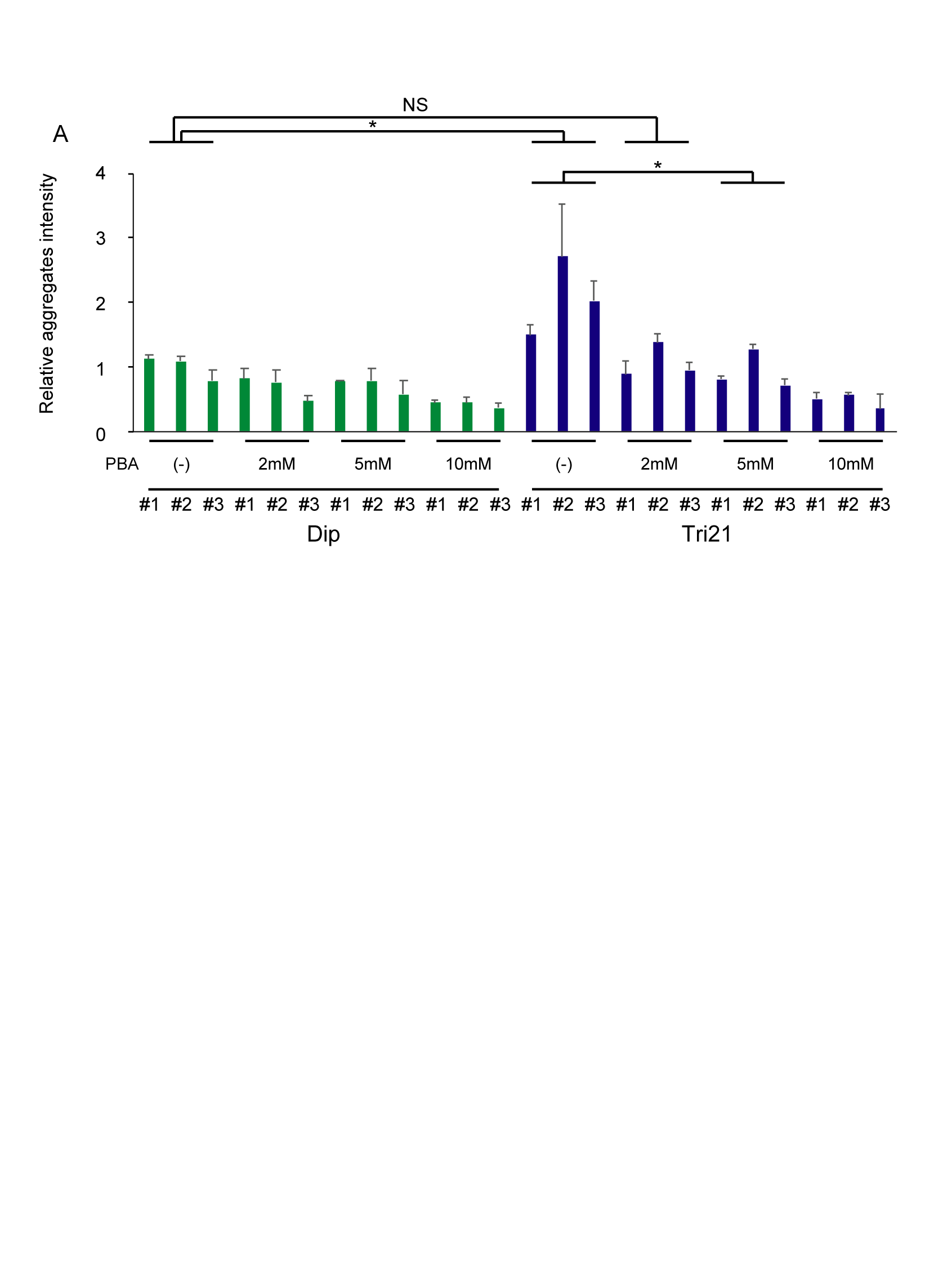

Supplement: S4 Fig — Data of three diploid and three trisomy 21 fibroblast cell lines are shown (n = 3 per cell line; original data in Fig 5D). *P < 0.05. PBA, sodium phenylbutyrate; Dip, diploid; Tri, trisomy; N.S., not significant. (TIF) [file pone.0219592.s004.tif]

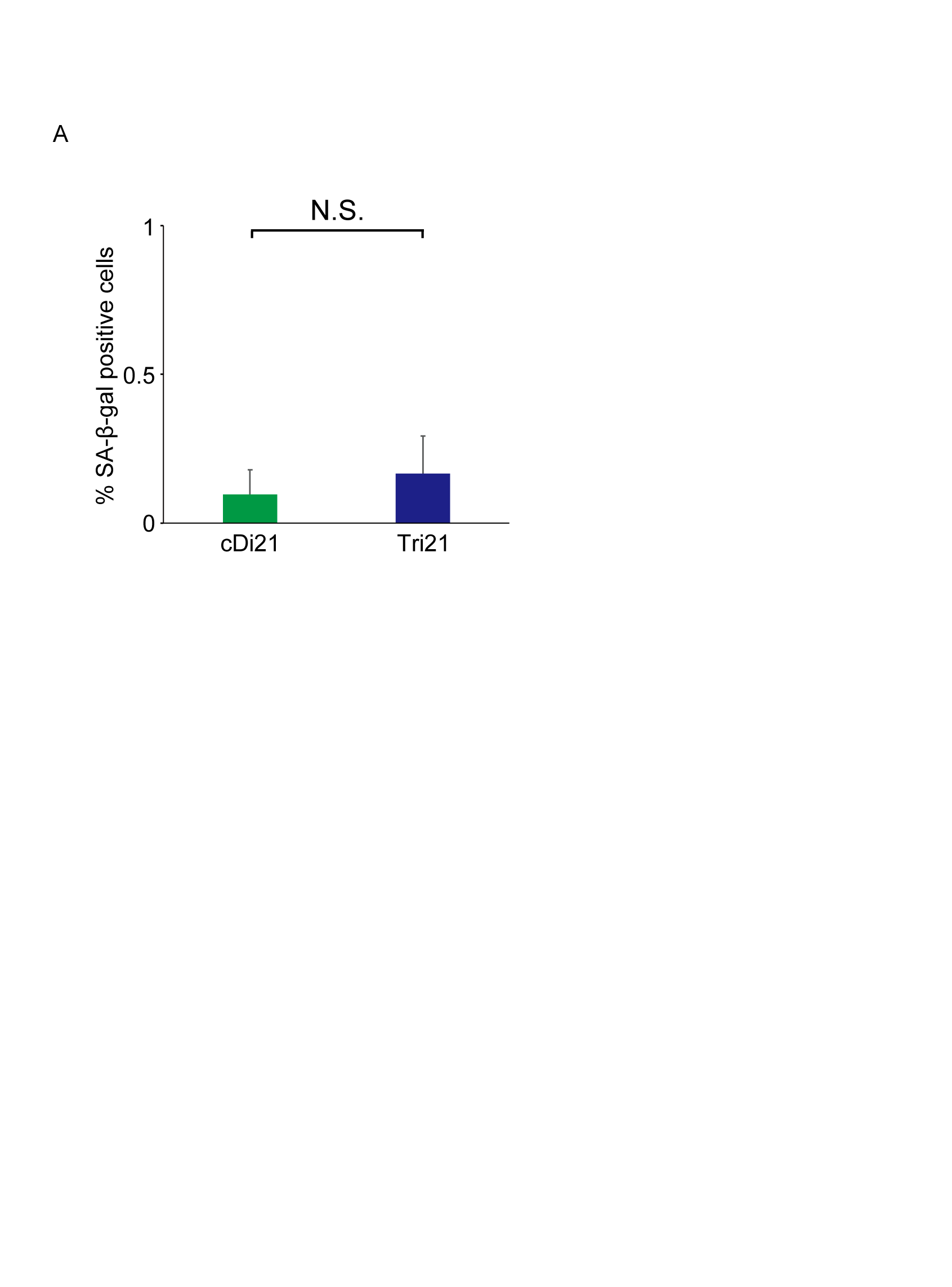

Supplement: S5 Fig — Percentages of SA-β-gal positive cells were calculated for undifferentiated iPSC lines (n = 4 per cell line). cDi21, corrected disomy 21 iPSCs; Tri21, trisomy21 iPSCs; N.S., not significant. (TIF) [file pone.0219592.s005.tif]
