# Supplementary material for: Elimination of protein aggregates prevents premature senescence in human trisomy 21 fibroblasts
Source: PLoS One. 2019 Jul 29;14(7):e0219592. doi: 10.1371/journal.pone.0219592 (PMC6663065; doi:10.1371/journal.pone.0219592)
Supplement: S1 Table — Information on sex and age at sample collection for each patient is shown. (DOCX) [file pone.0219592.s006.docx]

S1 Table. Characteristics of samples in the present study

| Patient | Sex | Age at sample collection |
| --- | --- | --- |
| Diploid #1 | M | neonate |
| Diploid #2 | M | neonate |
| Diploid #3 | M | neonate |
| Trisomy 21 #1 | M | 1y4m |
| Trisomy 21 #2 | M | 2y4m |
| Trisomy 21 #3 | F | 1m |
| Trisomy 21 #4 | M | 22d |
| Trisomy 18 #1 | M | 1m |
| Trisomy 18 #2 | F | 3y4m |
| Trisomy 18 #3 | M | 1m |
| Trisomy 13 #1 | F | 2d |
